# FDA approval of brensocatib: a new therapeutic breakthrough in non-cystic fibrosis bronchiectasis

**DOI:** 10.1097/MS9.0000000000004101

**Published:** 2025-10-14

**Authors:** Syeda Sunaina Hassan Gardezi, Nayyab Fatima, Usama Chaudhary, Maryam Irshad, Mahnoor Fatima, Syeda Shabih E Zahra Gardezi

**Affiliations:** a Nishtar Medical College, Nishtar Medical University, Multan, Pakistan; b Shaheed Ziaur Rahman Medical College, Rajshahi Medical University, Bogura, Bangladesh; c Department of Medicine, Multan Medical and Dental College, Multan, Pakistan

Non-cystic fibrosis bronchiectasis is a medical condition marked by the permanent and abnormal widening of the airways, especially the bronchi. This respiratory disorder arises from inflammation in the bronchi driven by neutrophils, frequent bacterial infections in the respiratory tract, and lasting lung damage, which eventually result in symptoms such as a persistent cough, sputum (often containing blood), shortness of breath, exhaustion, recurrent upper respiratory infections, and coughing up blood[1]. A complete global prevalence of bronchiectasis was reported in a recent meta-analysis that covered 15 previous studies, including over 437 million individuals. In the analysis, the worldwide prevalence of bronchiectasis was approximately 680 per 100 000 adults (95% CI: 634–727 per 100 000 adults). Diversity was reported when discussing the regional prevalence, with the estimation of 478 per 100 000 adults in the US, 886 per 100 000 adults in Korea, and 759 per 100 000 adults in China, underlining the increased global burden of bronchiectasis[2]. This editorial was written in accordance with TITAN 2025 checklist[3].

Historically, management approaches for non-cystic fibrosis bronchiectasis involved the use of long-term anti-inflammatory medications, mucolytic agents to break down mucus and clear the airways, oral and intravenous antibiotics to reduce the incidence of bacterial infections and exacerbations, inhaled bronchodilators and corticosteroids to alleviate airway obstruction, and lung surgery in cases of significant lung damage. However, none of these approaches specifically address the abnormal neutrophil activity that underlies the disease’s pathophysiology[4]. This highlights the necessity for anti-inflammatory treatments that can effectively reduce neutrophil-driven lung tissue damage without causing immunosuppression or contributing to antimicrobial resistance in patients.

In diseases characterized by neutrophilic inflammation, such as COPD and non-cystic fibrosis bronchiectasis, dysfunction in neutrophils leads to their excessive activity within lung tissue, resulting in the uncontrolled release of neutrophil serine proteases like neutrophilic elastase. This process disrupts normal ciliary function, triggers airway inflammation, and harms the lung’s airway epithelium. An increase in neutrophilic elastase levels in sputum is indicative of the severity and progression of bronchiectasis, correlating with a higher rate of exacerbations and a gradual decline in lung function[5]. Traditionally, neutrophilic elastase and phosphodiesterase 4 inhibitors have been employed to address neutrophilic inflammation in lung tissues; however, they have shown limited effectiveness due to adverse systemic side effects[6].

Amid ongoing challenges, the recent endorsement of brensocatib as the first-ever DPP-1 inhibitor by the US Food and Drug Administration marks a significant advancement in the treatment of non-cystic fibrosis bronchiectasis. Brensocatib is an oral medication taken once daily that selectively inhibits dipeptidyl peptidase 1 (DPP-1). As a dipeptidyl inhibitor, it interferes with the activation of neutrophil serine proteases, such as neutrophil elastase, during the maturation of neutrophils in the bone marrow, thereby reducing neutrophil elastase levels and directly diminishing neutrophilic inflammation in the airways. Brensocatib does not substantially affect immune function suppression, as demonstrated in Papillon–Lefèvre syndrome, where DDP-1 mutations lead to decreased neutrophil serine protease activity without contributing to immunodeficiency[7].

The clinical development of brensocatib involved randomized, double-blind, placebo-controlled clinical trials. The Phase 2 Willow Trial, which was a double-blind randomized placebo-controlled study, enrolled 256 adults with NCFB who had experienced at least two exacerbations in the prior year. The use of brensocatib in these patients resulted in the prolongation of time to first exacerbation (134 days compared to 67 days for the placebo group). Additionally, the annualized rates of exacerbations were found to be lower in the brensocatib groups compared to the placebo group. Notably, brensocatib led to a reduction in sputum neutrophil elastase activity. In this trial, mild side effects reported by patients with Papillon–Lefèvre syndrome included skin alterations resembling hyperkeratosis and mild periodontal disease[8].

Furthermore, the phase 3 ASPEN trial demonstrated that oral brensocatib effectively reduced pulmonary exacerbations, accompanied by enhancements in lung function, while also illustrating the drug’s efficacy and favorable safety profile, with mild side effects such as headache, rash, and minor upper respiratory tract infections[9]. Additionally, the trial revealed advancements in quality of life metrics as assessed by the St. George’s Respiratory Questionnaire. This underscores that the medication can be utilized not just for managing the disease, controlling symptoms, and preventing inflammation, but also for improving daily functioning[10]. The limitation of the ASPEN Phase 3 Trial is that these trials took place at the height of the COVID-19 pandemic, potentially resulting in a patient demographic that included a higher proportion of more critically ill individuals. While the outcomes for adolescents aligned with those of adults, the limited size of the adolescent group restricts definitive conclusions. Additionally, the trial was designed to assess the overall population rather than to identify differences in treatment effects among smaller, predefined subgroups[9].

When compared to traditional treatments for non-cystic fibrosis bronchiectasis, brensocatib offers several benefits. As a specific reversible DPP1 inhibitor, it exclusively focuses on the upstream activation of neutrophil serine proteases within the airways. Its safety profile is favorable, with typical side effects like skin and periodontal changes that are mild and manageable. Additionally, the reduction of neutrophil elastase in sputum linked to brensocatib serves as a significant pharmacodynamic marker, connecting the targeted therapy to clinical improvements, something not shown by earlier treatments[8]. Unlike long-term use of macrolides and antibiotics, brensocatib is a non-antibiotic and does not contribute to the development of antimicrobial resistance[11].

The most frequently reported side effects of brensocatib among study participants included skin rash, hyperkeratosis, dry skin, changes in gingival and periodontal health, upper respiratory infections, headaches, elevated blood pressure, and slight increases in liver enzyme levels. Rare side effects included hair loss and non-melanoma skin cancers. The live attenuated vaccines should be contraindicated while on brensocatib. The long-term safety profile of brensocatib requires further investigation in upcoming clinical trials, which will offer important insights into its extended use. Additionally, more research is necessary to evaluate the efficacy and safety of brensocatib in pregnant and breastfeeding individuals, as well as in children under 12 years old[12].

To sum up, brensocatib represents a notable milestone in the management of neutrophil-driven airway conditions. Its positive safety profile, innovative upstream mechanism, strong evidence from clinical research, and lack of antibiotic resistance make it an excellent option for treating non-cystic fibrosis bronchiectasis. Nevertheless, additional trials and clinical studies are essential, along with initiatives for global accessibility, to usher in a new era of neutrophil-modulating therapies that could benefit individuals around the globe.

## Data Availability

Not applicable.
